# Enhancing Light–Matter Interactions in MoS_2_ by Copper Intercalation

**DOI:** 10.1002/adma.202008779

**Published:** 2021-05-06

**Authors:** Chen Stern, Avraham Twitto, Rifael Z. Snitkoff, Yafit Fleger, Sabyasachi Saha, Loukya Boddapati, Akash Jain, Mengjing Wang, Kristie J. Koski, Francis Leonard Deepak, Ashwin Ramasubramaniam, Doron Naveh

**Affiliations:** ^1^ Faculty of Engineering Bar‐Ilan University Ramat‐Gan 52900 Israel; ^2^ Institute for Nanotechnology and Advanced Materials Bar‐Ilan University Ramat‐Gan 52900 Israel; ^3^ Nanostructured Materials Group International Iberian Nanotechnology Laboratory Avenida Mestre José Veiga s/n Braga 4715‐330 Portugal; ^4^ Electron Microscopy Group Defence Metallurgical Research Laboratory (DMRL) Hyderabad 500058 India; ^5^ Department of Chemical Engineering University of Massachusetts Amherst MA 01003 USA; ^6^ Department of Chemistry University of California Davis Davis CA 95616 USA; ^7^ Department of Mechanical and Industrial Engineering University of Massachusetts Amherst MA 01003 USA

**Keywords:** copper intercalation, MoS _2_, photodetectors

## Abstract

The intercalation of layered compounds opens up a vast space of new host–guest hybrids, providing new routes for tuning the properties of materials. Here, it is shown that uniform and continuous layers of copper can be intercalated within the van der Waals gap of bulk MoS_2_ resulting in a unique Cu–MoS_2_ hybrid. The new Cu–MoS_2_ hybrid, which remains semiconducting, possesses a unique plasmon resonance at an energy of ≈1eV, giving rise to enhanced optoelectronic activity. Compared with high‐performance MoS_2_ photodetectors, copper‐enhanced devices are superior in their spectral response, which extends into the infrared, and also in their total responsivity, which exceeds 10^4^ A W^−1^. The Cu–MoS_2_ hybrids hold promise for supplanting current night‐vision technology with compact, advanced multicolor night vision.

## Graphical abstract

Intercalation of uniformly distributed copper into the van der Waals gap of vertically aligned MoS_2_ grown by chemical vapor deposition improves photodiode device performance. The intercalated MoS_2_ remains semiconducting and possesses a unique plasmon resonance at an energy of ≈1 eV. Remarkably enhanced photoresponse from the ultraviolet to near‐infrared regime is presented.

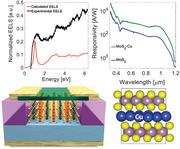

## 1 Introduction

Progress in the research of 2D layered compounds has continued at a sustained pace over the last decade with the discoveries of new compounds,^[^ ^1^, ^2^ ^]^ physical phenomena,^[^ ^3^, ^4^ ^]^ and technological advancements.^[^ ^5^, ^6^ ^]^ Intercalation chemistry in layered compounds, where the van der Waals (vdW) gap of the host compound is filled with guest (intercalant) atoms^[^ ^7^ ^]^ or molecules,^[^ ^8^ ^]^ is a cornerstone in many processes and forms the basis of modern energy storage devices.^[^ ^9^, ^10^ ^]^ In some cases, the intercalation process is designed to modify the properties of the host material,^[^ ^11^ ^]^ whereas in other cases, intercalation produces a continuous 2D material of interest, that would be hard to realize without the template of the confining environment of the vdW gap. For example, 2D gallium and indium that are grown by atomic intercalation at the interface of SiC and graphene, show potential for superconducting devices, topological phenomena, and advanced optoelectronic properties.^[^ ^12^ ^]^ Another example of such intercalated layers are zero‐valent metals and semiconductors, demonstrated in several host layered compounds.^[^ ^13^, ^14^ ^]^ In the case of ionic intercalation, self‐intercalation of Cu^+1^ has improved the electronic transport in layered metal–chalcogen compounds.^[^ ^15^ ^]^ These techniques have resulted in a vast array of chemically tunable behaviors including ambipolar optoelectronics in SnS_2_,^[^ ^16^ ^]^ chemochromism in MoO_3_,^[^ ^17^ ^]^ tunable enhanced transparency in Bi_2_Se_3_,^[^ ^18^, ^19^ ^]^ enhanced catalytic and energy storage behaviors in oxides,^[^ ^20^, ^21^ ^]^ tunable mechanical properties,^[^ ^22^ ^]^ and enhanced thermoelectric performance in bismuth telluride.^[^ ^23^, ^24^ ^]^ The ability to chemically tune material properties—including optical and electronic properties—through intercalation opens a vast space of new physical and chemical behaviors. As MoS_2_ has already achieved significant interest for its sizable photoresponse, further intensifying light–matter interactions and amplifying the response in the near‐infrared (NIR) is a very attractive goal. Here, we demonstrate intercalation of uniformly distributed planar layers of copper into devices of vertically aligned MoS_2_ (VA‐MoS_2_) grown by chemical vapor deposition (CVD).^[^ ^25^, ^26^, ^27^ ^]^ The intercalated material remains semiconducting and displays an enhanced photoresponse that we attribute to a hybrid plasmon of Cu–MoS_2_. The Cu–MoS_2_ devices show a broad spectral response with a maximum value of 10^4^ A W^−1^ and extending into the NIR range, enabling potential applications in compact, multicolor night‐vision technology.

## 2 Results and Discussions

**Figure 1:**
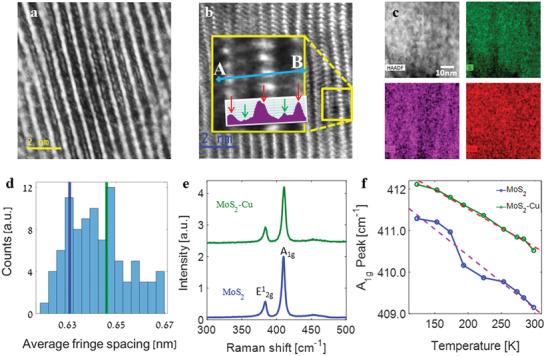
Structural characterization of VA‐MoS_2_ intercalated with zero‐valent Cu atoms. a) HRTEM image displaying an additional layer due to intercalation of Cu within the vdW gaps. b) STEM‐HAADF image showing a *z*‐contrast image with the inset showing the magnified microscopy image of the area enclosed by the yellow square along with intensity profile along the line AB in the inset, with the layer of Mo‐atoms appearing with higher intensity and Cu‐atoms in between the layer of bright Mo‐atoms, showing up with lower intensity. c) HAADF microscopy image along with elemental distribution maps collected from the energy‐dispersive spectroscopy. d) Distribution and mean value of interlayer spacing between VA‐MoS_2_ planes before (blue line) and after (green line) Cu intercalation. e) Raman spectra of 2H‐MoS_2_ structure before (blue spectrum) and after (green spectrum) Cu intercalation. f) Temperature‐dependent evolution of the peak of A_1g_ Raman mode of MoS_2_ before (blue) and after (green) Cu intercalation.

Cu–MoS_2_ hybrids were prepared by zero‐valent intercalation^[^ ^28^ ^]^ on CVD‐grown VA‐MoS_2_^[^ ^27^ ^]^ (see Section 4 for details) followed by deposition of top indium tin oxide (ITO) contact (see Figure 4), and a thin electron transparent lamella of the cross section of the device structure for inspection was prepared by the focused ion beam (FIB). The close‐packed arrangement of copper atoms in MoS_2_ host and their impact on the structure are inferred from the high‐resolution transmission electron microscopy (HRTEM) and Raman spectra displayed in **Figure** **1**. Figure 1a displays an HRTEM image of MoS_2_ after intercalation of Cu atoms under the parallel beam illumination condition, showing the presence of fringe contrast coming from parallel sets of planes. The presence of an additional distinct layer within the vdW gap of MoS_2_ suggests that copper atoms organize in close packing within the vdW space. The slight distortions of the layer planes (Figure 1a,b) are suggestive of some islanding of the copper within the host, consistent with a Daumas–Herold mechanism of intercalation.^[^ ^29^ ^]^ The average interplanar spacing is found to be greater than the expected interplanar spacing for regular, intercalation‐free MoS_2_, as seen from the distribution and mean value of the measured interlayer fringe spacing (Figure 1d). The average fringe spacing in the intercalated sample increases from 0.6309 to 0.6458 nm—an increase of 0.0149 nm. This layer expansion of 2.3% is significant and points to a substantial expansion the of MoS_2_ interlayer spacing due to Cu intercalation. This expansion is also illustrated in the high‐magnification image in Figure 1a,b, which clearly shows the presence of an additional plane between two consecutive MoS_2_ (0002) planes. Figure 1c shows a high‐angle annular dark‐field scanning transmission electron microscopy (STEM‐HAADF) image of a small region within the intercalated VA‐MoS_2_ sample and its corresponding colored TEM energy‐dispersive X‐ray spectrum (EDX) mapping of Cu. The Cu is found to be distributed throughout the host material. The presence of Cu in MoS_2_ is further confirmed from the Cu peaks seen in the EDS spectra (Figure S2, Supporting Information) acquired from the regions shown in Figure 1c. The copper concentration is found to be about 11 atomic percent, yielding a stoichiometry of Cu_0.11_MoS_2_.

After Cu intercalation there are now more conductive atoms within the host MoS_2_ structure that can play a key role in enhancing the electrical and optical properties, as discussed in detail later. Interestingly, the Raman spectra taken before and after intercalation show only small differences (Figure 1e). Spectra taken at a parallel geometry between the laser line and the *c*‐axis of the crystal with linearly polarized light display the expected modes of 2H‐MoS_2_, including an E^1^_2g_ mode at ≈383 cm^−1^ that corresponds to an in‐plane stretch and an A_1g_ mode at ≈410 cm^−1^ that corresponds to an out‐of‐plane breathing mode.^[^ ^30^ ^]^ With intercalation of Cu, MoS_2,_ Raman modes show stiffening with an increase of ≈2 cm^−1^ for the A_1g_ mode (Figure 1f). The increase in the Raman wavenumber shift with intercalation is consistent with optical phonon stiffening and has been observed in other intercalated systems.^[^ ^22^, ^31^ ^]^ The thermal coefficient of the A_1g_ mode of bare MoS_2_ (0.0127 cm^−1^ K^−1^) agrees well with previous reports.^[^ ^32^ ^]^ The thermal coefficient of the Cu‐intercalated samples (0.0091 cm^−1^ K^−1^) is ≈28% lower than the pristine MoS_2_, and consequently the measured thermal conductivity decreases proportionally after the intercalation (see Supporting Information).^[^ ^33^ ^]^

**Figure 2:**
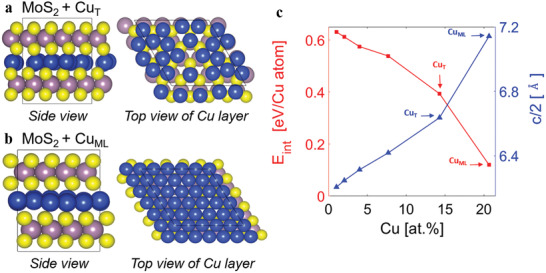
a,b) Structural models of a partial Cu layer (Cu_T_) (a) and a Cu(111) monolayer (Cu_ML_) (b) intercalated within a single vdW gap of bulk MoS_2_. Unit cells are indicated by solid lines; one of the layers has been removed in the top views to show the intercalated Cu layers clearly. In (b), the supercell consists of a 5 × 5 monolayer of Cu(111) which is nearly commensurate with a 4 × 4 cell of MoS_2_; in (a) one Cu atom was initially inserted at every hollow site (between three S atoms) and allowed to relax, forming disordered 2D clusters. c) Energy per Cu atom (*E*_int_) required for intercalation into the vdW gap (red line) and the resulting interlayer separation (*c*/2; blue line) both as functions of Cu concentration. Specific concentrations corresponding to the Cu_ML_ and Cu_T_ models are noted in the figure.

Density functional theory (DFT) calculations were performed to gain further insights into the structural and electronic properties of the Cu‐intercalated MoS_2_ structures. At low concentrations, intercalated Cu atoms bind to basal‐plane sulfur atoms and are tetrahedrally coordinated.^[^ ^34^, ^35^ ^]^ With increasing concentration though, the Cu atoms tend to cluster spontaneously within the vdW gap (**Figure** **2**a), ultimately forming slightly corrugated Cu (111) monolayers (Figure 2b). This computationally derived picture is in agreement with the experimental observations in Figure 1a,b, wherein we see partial to complete layers of Cu intercalated within the MoS_2_ vdW gap. The driving force for clustering can be quantified by the energetic cost of Cu intercalation (per atom), *E*_int_, defined as

(1) $$E_{\mathrm{int}}=\frac{1}{n_{x}}\;\;\left(E_{{\text{Cu}}_{x}{\text{MoS}}_{2}}-E_{{\text{MoS}}_{2}}-n_{x}E_{\text{Cu},\text{bulk}}\right)$$

where *x* is the atomic percentage of intercalated Cu in the Cu*_x_* MoS_2_ structure and *n_x_* is the total number of Cu atoms; $E_{{\text{Cu}}_{x}{\text{MoS}}_{2}}$, $E_{{\text{MoS}}_{2}}$, and *E*_Cu,bulk_ are the 0 K DFT energies of Cu*_x_* MoS_2_, pristine MoS_2_, and a single Cu atom (bulk FCC structure), respectively. As seen from Figure 2c, the intercalation energy progressively decreases with increasing Cu intercalation, going from nearly 0.63 eV per atom for a single Cu atom to about 0.12 eV per atom for a complete monolayer. An intuitive physical picture for this observation may be proposed as follows. When Cu is intercalated into the vdW gap, there is an energetic cost associated with expanding the gap to accommodate the Cu atoms as well as an energetic cost associated with activating the fully coordinated, inert basal plane S atoms to form Cu—S bonds. Thus, the intercalation energy is initially high. With progressive Cu intercalation, first, the cost of destabilizing the vdW interaction between MoS_2_ layers is partially compensated by interactions between clusters of Cu atoms (partial layers) and the MoS_2_ layers. Second, and more important, as Cu atoms begin to cluster, we find that the number of Cu—S bonds are reduced and, consequently, the S atoms of the MoS_2_ basal planes are less destabilized. The ultimate limit of a “cluster” is a complete Cu (111) monolayer, which only has weak chemical interactions (bonding) with the MoS_2_ layers but does have significant charge‐transfer interactions, as will be discussed later. Finally, we also note the systematic expansion of the interlayer spacing (Figure 2c) from 6.17 Å in pristine MoS_2_ to 7.14 Å for intercalation of a Cu monolayer. While this interlayer expansion is larger than in experiments, it should be noted that the DFT models allow for complete relaxation of the structure whereas, in practice, the VA‐MoS_2_ layers are confined by the Si wafer.

**Figure 3:**
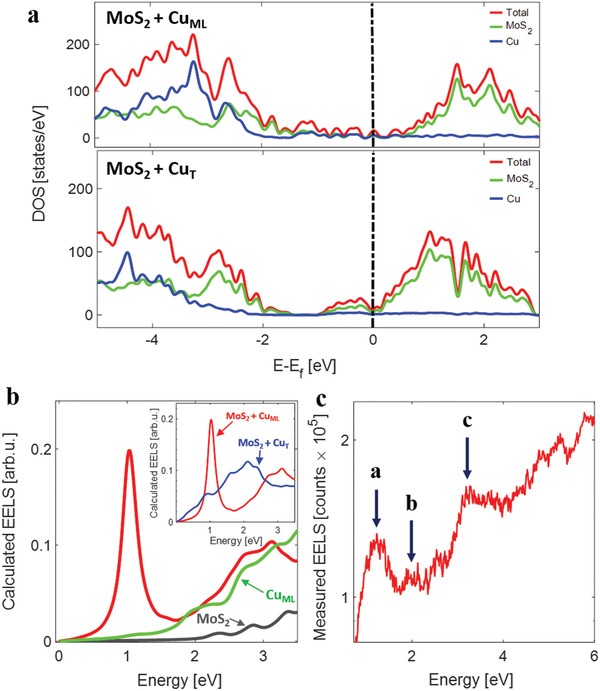
a) Density of states (DOS) of MoS_2_ + Cu_ML_ and MoS_2_ + Cu_T_ models calculated with the HSE functional; the total DOS (red lines) is further decomposed into contributions from MoS_2_ (green lines) and Cu (blue lines). b) Calculated EELS spectrum of the MoS_2_ + Cu_T_ model (red line), bulk MoS_2_ (gray line) and the isolated Cu_ML_; the inset compares the calculated EELS of the MoS_2_ + Cu_ML_ and MoS_2_ + Cu_T_ models. c) Low‐loss EELS spectra obtained from the Cu‐intercalated sample after removing the zero line peak.

The electronic structures and optical properties of the Cu*_x_* MoS_2_ models are displayed in **Figure** **3**a,b. Upon examining the electronic density of states in Figure 3a, we find that the intercalated Cu introduces electronic states near the conduction band edge of MoS_2_ and shifts the Fermi level close to the conduction band edge (*n* doping). At lower concentrations of intercalated Cu—associated with small clusters and some degree of Cu—S bonding—these additional electronic states form a broad continuum near the conduction band edge; once the intercalated Cu forms a complete monolayer, these additional states appear as sharp resonances within the MoS_2_ bandgap. The calculated EELS spectrum in Figure 3b reveals the emergence of a new plasmonic peak at ≈1.1 eV in the Cu‐monolayer intercalated structures (MoS_2_ + Cu_ML_ model); this plasmon peak is not present either in bulk MoS_2_ or in a Cu (111) monolayer, and is unique to the intercalated structure. Moreover, this sharp plasmon peak is not present in the partially intercalated structure (MoS_2_ + Cu_T_), which only displays an overall enhancement in the electron energy loss spectroscopy (EELS) in the low‐energy range (see inset of Figure 3b). We attribute the sharp plasmonic feature in the fully intercalated structure to optical transitions involving the numerous resonant gap states seen in Figure 3a. As the actual VA‐MoS_2_ samples consist of regions of complete, partial, or no Cu intercalation, the measured low‐energy EELS spectrum is expected to reflect a weighted average of the calculated spectra from the partial and fully intercalated samples.

Figure 3c displays the measured low‐loss EELS spectra from the Cu‐intercalated MoS_2_ sample and clearly reveals the emergence of new peaks plasmonic peaks as predicted by the density functional theory (DFT) calculations. The measured EELS spectrum displays three distinct peaks corresponding to: a) a hybrid MoS_2_–Cu excitation at ≈1.3eV in correspondence with the MoS_2_–Cu_ML_ model; b) a smaller peak at ≈2 eV in correspondence with the MoS_2_–Cu_T_ model; and c) a broad peak at ≈3.5 eV corresponding to the bare MoS_2_. The interpretation of the spectra, in conjunction with the DFT models, provides a picture wherein the intercalated Cu organizes within the vdW gap of MoS_2_ in large (nearly continuous) domains as well as smaller patches. Intercalation, in general, can result in new EELS peaks from the appearance of new surface plasmons, bulk plasmons, or interband transitions. The optical doublet of MoS_2_ from excitonic transitions^[^ ^36^, ^37^ ^]^ identified as A and B excitons, appears to be suppressed with intercalation. This suppression of optical modes has been observed in Cu‐intercalated Bi_2_Se_3_.^[^ ^38^ ^]^ The peak at ≈3eV is seen in MoS_2_ and is attributed to excitonic transitions, specifically the C‐exciton of MoS_2_.^[^ ^36^ ^]^ This peak is attributed to transitions between the Γ−Λ point of the Brillouin zone ^[^ ^37^ ^]^ and does not vanish with Cu intercalation. The emergence of new plasmon peaks of intercalated metals, particularly copper, have been observed previously in other materials systems.^[^ ^17^, ^38^ ^]^ For example, a new EELS peak was seen at 4.3 eV in germanium‐intercalated Si_2_Te_3_ that was attributed to a new surface plasmon, bulk plasmon, or interband transition.^[^ ^17^ ^]^ Cu intercalation in Si_2_Te_3_ resulted in a shift of the bulk plasmon peak and the appearance of a new shoulder edge on the bulk plasmon peak.^[^ ^31^ ^]^ Cu intercalation in solvothermally grown Bi_2_Se_3_ also has shown the appearance of new plasmon modes and the broadening of existing peaks in the low‐loss and higher regions.^[^ ^38^ ^]^ Intrigued by the emergence of these new plasmons, we examine next the optoelectronic activity of the modified semiconductor, in particular, as photodiode devices.

**Figure 4:**
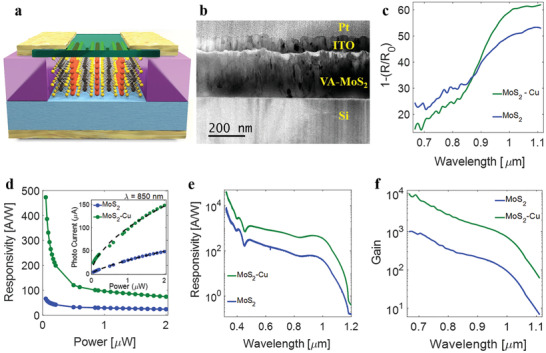
a) Graphical illustration of the cross‐section of a VA‐MoS_2_ (yellow and black atoms)–Si (light blue) heterostructure photodiode device intercalated by Cu (red atoms). b) Cross‐section transmission electron microscopy (TEM) image of the device. c) Absorption versus wavelength of the bare‐MoS_2_ device (blue) and the Cu‐intercalated MoS_2_ device (green). d) Responsivity as a function of incident power intensity of the bare‐MoS_2_ device (blue) and the Cu‐intercalated MoS_2_ device (green) under illumination of 850 nm at a reverse bias of −2 V. Inset: Dependence of photocurrent on incident power at wavelength of 850 nm. e) Responsivity versus wavelength of the bare‐MoS_2_ device (blue) and the Cu‐intercalated MoS_2_ device (green). f) Gain versus wavelength of the bare‐MoS_2_ device (blue) and the Cu‐intercalated MoS_2_ device (green).

**Figure** **4**a displays a schematic representation of a Cu‐intercalated VA‐MoS_2_ photodiode that comprises a Pd back contact to a P–Si wafer and an ITO top contact (see Section 4 for fabrication details). A cross‐sectional TEM image of the device structure is displayed in Figure 4b. The microscopy image clearly shows demarcations between the different layers present in the device structure: a layer of protective platinum on the top, which was deposited during the FIB sample preparation, followed by a layer of ITO, followed by the active layer of 70 nm VA‐MoS_2_ grown on the Si substrate. The total absorbance of the devices is displayed in Figure 4c. The bare MoS_2_ device has optical absorptions between 20–50% over the range of 0.7–1.1 µm wavelengths respectively. Cu intercalation further enhances the NIR absorption of the device up to ≈60%. The photoresponse and photocurrent (at 850 nm) of VA‐MoS_2_ and Cu‐intercalated VA‐MoS_2_ are displayed in Figure 4d,e, respectively. The responsivity of the MoS_2_\Si diode peaks at ≈ 66 A W^−1^  whereas the responsivity of the Cu‐intercalated device peaks at ≈500 A W^−1^. The responsivity of 66 A W^−1^ is higher than most photodetectors based on MoS_2_\Si heterojunctions of this type.^[^ ^39^, ^40^, ^41^, ^42^, ^43^, ^44^, ^45^ ^]^ Remarkably, intercalation of Cu improves the responsivity of the photodiode by an order of magnitude (Figure 4e) over a wide spectral range of ≈0.5–1.1 µm. At greater light intensities, the responsivity of the Cu‐intercalated device decays^[^ ^46^ ^]^ but still remains higher than that of the unintercalated device by a factor of three (Figure 4d).

The spectral response displayed in Figure 4e and the absorbance are related to the internal quantum efficiency (IQE), η_*i*_, by the relation $\eta_{i}\;(\lambda )=\frac{R(\lambda )hc}{\lambda \rho (\lambda )}\;$, where λ is the wavelength of incident light, *h* is Planck's constant, *c* is the speed of light, and ρ(λ) is the absorbance; $R\;(\lambda )=\frac{I_{ph}(\lambda )}{P(\lambda )}\;$ is the spectral responsivity, where *I*_ph_(λ) is the photocurrent, and *P*(λ) is the power spectrum. Since the upper bound of IQE is 1, we attribute the excess of electrons collected per photon to a photoconductive gain. The gain (Figure 4f) at the 0.7–1.1 µm wavelength range attains peak values of ≈1000 and ≈10 000 for the pristine and Cu‐intercalated devices, respectively. We attribute the high gain to defects in VA‐MoS_2_ that trap electrons for durations that are longer than the transit time of holes.^[^ ^55^ ^]^ **Table** **1** outlines a comparative survey of Si–MoS_2_ photodetector device performances. The maximum photoresponsivity of our devices (4.2  × 10^4^ and 8.2  × 10^3^ for Cu:VA‐MoS_2_ and for the VA‐MoS_2_, respectively) is found to be higher than most other devices. In addition to the responsivity peak value, the broad spectral response features high average values of 161 A W^−1^ before and 830 A W^−1^ after Cu intercalation, featuring a plateau in the spectral range of 500–847 nm. The Cu intercalation induces a photoconductive gain at a cost of some increase in response time; nevertheless, the photodetectors remain relatively fast (see Table 1). With the addition of Cu via intercalation, the additional gain is almost homogeneously distributed over the entire spectrum. Since the differences in optical absorption and electrical conductivity of the Cu‐intercalated devices relative to bare MoS_2_ are small, we attribute the photoresponse enhancement to the efficient ionization of zero‐valent copper by photocarriers. This enhancement can be technologically beneficial for night‐vision image intensifiers, working at low‐light levels.^[^ ^56^ ^]^ Traditionally, image intensifiers are based on intensifying tubes that convert scene photons to electrons on a photocathode; the electrons are multiplied via a multi‐channel plate (MCP) and are accelerated to produce an enhanced image of the scene on a phosphorus screen. The intercalation‐enhanced photodetectors may be good candidate technology for the core elements of vis to NIR image intensifiers, replacing bulky tubes with compact color vision. The high performance within the NIR part of the spectrum could allow for extra imaging capabilities under low‐light (photon counting) scenarios, even on moonless nights due to the atmospheric night glow phenomena. In addition, the NIR band is most suitable for imaging and analysis of high vegetation terrain (e.g., forests, agricultural fields).

**Table 1:** Comparison of the characteristics of Si–MoS_2_ photodetectors reported in this work and those in previous reports

Device	Responsivity [A W^−1^]	Wavelengh [nm]	Response time [rise/fall]	Ref.
Si/VA‐MoS_2_	322.3–4.3	485–1100	5.2/1.2 µs	This work
	max: 8.26 × 10^3^	325		
Si/Cu–VA‐MoS_2_	1301–46	485–1100	16.7/11.2 µs	This work
	max: 4.2 × 10^4^	323		
Si/VA‐MoS_2_	8.75	580	10/19 µs	^[^ ^39^ ^]^
Si/VA‐MoS_2_	0.3	808	3/40 µs	^[^ ^40^ ^]^
Si/VA‐MoS_2_	0.908	808	56/825 ns	^[^ ^41^ ^]^
Si/VA‐MoS_2_	7.37	532	—	^[^ ^47^ ^]^
Si/VA‐MoS_2_	0.03	455	38.78/43.07	^[^ ^48^ ^]^
Si/VA‐MoS_2_	0.654	980	2.1/173.8 µs	^[^ ^49^ ^]^
Si/VA‐MoS_2_	49.31	800	80/79 ms	^[^ ^50^ ^]^
Single‐layer MoS_2_ FET	880	561	4/9 s	^[^ ^51^ ^]^
Si/single‐layer MoS_2_	7.2	365	50/50 ms	^[^ ^43^ ^]^
Si/few‐layers‐MoS_2_	76.1	660	>50/48.9 s	^[^ ^52^ ^]^
Si/thin‐film‐MoS_2_	23.1	780	21.6/65.5 µs	^[^ ^44^ ^]^
Si/multilayer MoS_2_	11.9	650	30.5/71.6 µs	^[^ ^53^ ^]^
Si/MoS_2_ Q.D.	2.8	514	—	^[^ ^54^ ^]^

## 3 Conclusions

We have studied the impact of Cu intercalation on the electrical and optical properties of MoS_2_ through a combination of materials characterization, theoretical calculations, and device measurements. Interestingly, Cu‐intercalated MoS_2_ remains semiconducting even at high densities of Cu intercalation. Structurally, intercalation results in a 2.3% expansion of the MoS_2_ interlayer spacing, accompanied by stiffening of the Raman *A*_1_ mode and higher thermal conductivity. The Cu‐intercalated MoS_2_ samples were implemented in fast, spectrally broad, highly responsive photodiodes. Relative to pristine MoS_2_ devices, the Cu‐intercalated devices showed over an order of magnitude enhancement in photoresponse over a range of 0.5–1.1 µm. With a photoconductive gain reaching 10 000, the devices are promising candidates for night‐vision imaging, leading to significant improvements over the photomultiplier‐phosphor plate technology.

## 4 Experimental Section

### Sample Preparation

The fabrication of CVD‐grown MoS_2_–Si diodes is described in detail in ref. ^[^ ^27^ ^]^. Zero‐valent Cu atoms were intercalated into the vdW gaps between the layers of VA‐MoS_2_ by a wet‐chemical process.^[^ ^13^ ^]^ Briefly, 0.01 g of tetrakisacetonitrile copper hexafluorophosphate (Millipore‐Sigma) was added to 5 mL acetone (Millipore‐Sigma) in a round‐bottom flask attached to a Liebig reflux condenser. The solution was brought almost to reflux at 48 °C. The substrate was placed into the solution in the round‐bottom flask. The solution was allowed to sit just below reflux for 4 h, whereupon it was removed and rinsed with acetone several times. ITO and metal contacts were finally deposited after patterning with e‐beam lithography.

### Sample Characterization—Raman Spectroscopy

Raman spectra were obtained using a Horiba Scientific Labram HR Evolution equipped using 532 nm solid state excitation laser and an optical microscope. The laser excitation propagated parallel to the crystal *c*‐axis with linear polarization. A 50× objective lens was used to focus the laser and collect the Raman scattered light, and an 1800 lines per mm grating was chosen for spectrum acquisition.

### Sample Characterization—FIB, HRTEM, and STEM‐EDS

Site‐specific, cross‐sectional FIB prepared TEM lamellae were imaged using a Probe corrected FEI Titan G2 ChemiSTEM TEM equipped with a Super‐X EDX system, which comprised four windowless silicon drift detectors of 120 mm^2^ size, having an overall energy resolution better than 140 eV. The microscope was mounted with a Gatan 994 UltraScan 4K CCD camera, and the system had a point resolution better than 0.24 nm in the TEM mode and 80 pm in the STEM mode at 200kV accelerating voltage. The samples were imaged both in TEM and STEM modes as well as analyzed using STEM‐EDS. To avoid any stray Cu signals in the EDS spectra, appropriate care was taken and molybdenum grids, rings, and clips were used for loading the sample in the TEM holder.

The EDS data were acquired and analyzed using the Bruker QUANTAX Esprit 1.9 software. The elemental quantification was performed using the Cliff–Lorimer method with the same software.

EELS was performed using a double‐corrected FEI Titan Themis 60–300 kV equipped with a gun monochromator and a Gatan GIF Enfinium Fast dual EELS spectrometer, providing an energy spread of less than 0.19 eV at 60 kV. The spectra were acquired with an energy dispersion of 0.01 eV per channel, using 2.5 mm entrance aperture, and with 0.005 s exposure integrated over 2 s.

### Sample Characterization—Photoconductivity Measurements

The monochromatic measurements were carried out at ambient conditions under illumination of a collimated 850 nm light‐emitting diode (LED) (as discussed in the Supporting Information). Spectral responsivity and photocurrent were obtained by connecting the devices to an external detector socket of a Thermo Fisher Scientific Nicolet‐iS50R. In order to obtain the Quartz‐Halogen source black‐body radiation curve, the source was measured using a DTGS detector. The device photocurrent spectrum was normalized to the source black‐body curve to obtain the device's responsivity curve.

### Sample Characterization—Computational Methods

Density functional theory (DFT) calculations were performed using the Vienna Ab initio Simulation Package (VASP; version 5.4.1).^[^ ^57^, ^58^ ^]^ Core and valence electrons were described using the projector‐augmented wave method^[^ ^59^, ^60^ ^]^ and the Perdew–Burke–Ernzerhof generalized‐gradient approximation^[^ ^61^ ^]^ was used to describe electron exchange and correlation. The kinetic energy cutoff was set to 400 eV and Gaussian smearing of 0.05 eV was used for integrations over the Brillouin zone. The conjugate‐gradient algorithm was used for structural optimizations of all DFT models with a tolerance of 0.01 eV per Å. During structural optimization, both atomic positions and cell vectors were relaxed. To model the intercalation of Cu atoms in bulk 2H‐MoS_2_, 4  ×  4  ×  1 MoS_2_ supercells were employed; this supercell was nearly commensurate with a 5  ×  5 Cu (111) monolayer. The DFT‐D3 method of Grimme et al.^[^ ^62^ ^]^ was employed to include vdW interactions between MoS_2_ layers. A sufficiently dense 4  ×  4  ×  3 Γ‐centered *k*‐point mesh was used to sample the Brillouin zones of the supercells. As semilocal functionals underestimate fundamental gaps, the hybrid Heyd–Scuseria–Ernzerhof (HSE06)^[^ ^63^ ^]^ functional was employed to calculate electronic structure and optical properties; PBE‐relaxed Cu*_x_* MoS_2_ structures were used in these calculations as the computational cost of structural relaxation with HSE is prohibitive.

## Conflict of Interest

The authors declare no conflict of interest.

## Author Contributions

C.S. performed CVD growth; C.S. fabricated the devices; K.J.K. performed the intercalation process; C.S., R.S., and A.T. performed optical/electrical measurements; D.N., S.S., K.J.K., L.B., A.T., C.S., F.L.D. and R.S. analyzed the results; Under supervision of F.L.D., S.S. performed TEM/HRTEM, STEM‐HAADF, EDS studies and LB performed monochromated‐EELS measurements; A.T., R.S., and D.N. conducted the FTIR measurements; Y.F. performed FIB; A.R. and A.J. performed the theoretical calculations and simulations; D.N. conceived and supervised the study; all authors read and agreed with the manuscript.

## Supporting information

Supporting Information

## Data Availability

The data that support the findings of this study are available from the corresponding author upon reasonable request.
