# Supplementary material for: Enhancing Light–Matter Interactions in MoS2 by Copper Intercalation
Source: Adv Mater. 2021 May 6;33(23):2008779. doi: 10.1002/adma.202008779 (PMC11469038; doi:10.1002/adma.202008779)
Supplement: Supplementary file 1 — Supporting Information [file ADMA-33-2008779-s001.pdf]

© 2021 Wiley-VCH GmbH

ADVANCED MATERIALS

Supporting Information

for *Adv. Mater.,* DOI: 10.1002/adma.202008779

Enhancing Light–Matter Interactions in MoS_2 by Copper Intercalation

*Chen Stern, Avraham Twitto, Rifael Z. Snitkoff, Yafit Fleger, Sabyasachi Saha, Loukya Boddapati, Akash Jain, Mengjing Wang, Kristie J. Koski, Francis Leonard Deepak, Ashwin Ramasubramaniam, and Doron Naveh**

Supporting Information

Enhancing Light–Matter Interactions in MoS_2 by Copper Intercalation

Chen Stern,[1,2] Avraham Twittto,[1,2] Rifael Z. Snitkoff,[1,2] Yafit Fleger,[2] Sabyasachi Saha,[4,7] Loukya Boddapati,[4] Akash Jain,[5] Mengjing Wang,[3] Kristie J. Koski,[3] Francis Leonard Deepak,[4] Ashwin Ramasubramaniam,[6] and Doron Naveh [1,2,*]

[1] Faculty of Engineering, Bar-Ilan University, Israel.
[2] Institute for Nanotechnology and Advanced Materials, Bar-Ilan University, Israel.
[3] Department of Chemistry, University of California Davis, USA.
[4] Nanostructured Materials Group, International Iberian Nano Technology Laboratory, Portugal.
[5] Department of Chemical Engineering, University of Massachusetts, Amherst, MA 01003, USA.
[6] Department of Mechanical and Industrial Engineering, University of Massachusetts, Amherst, MA 01003, USA.
[7]Electron Microscopy Group, Defence Metallurgical Research Laboratory (DMRL), Hyderabad, India – 500058.
[*] Corresponding author: doron.naveh@biu.ac.il

TEM Structural Characterization:

Measurements of the c-plane spacing (**Figure 1d**) were performed be averaging the distance across bright fringes divided by the number of intensity peaks, as demonstrated in **Figure S1** below.

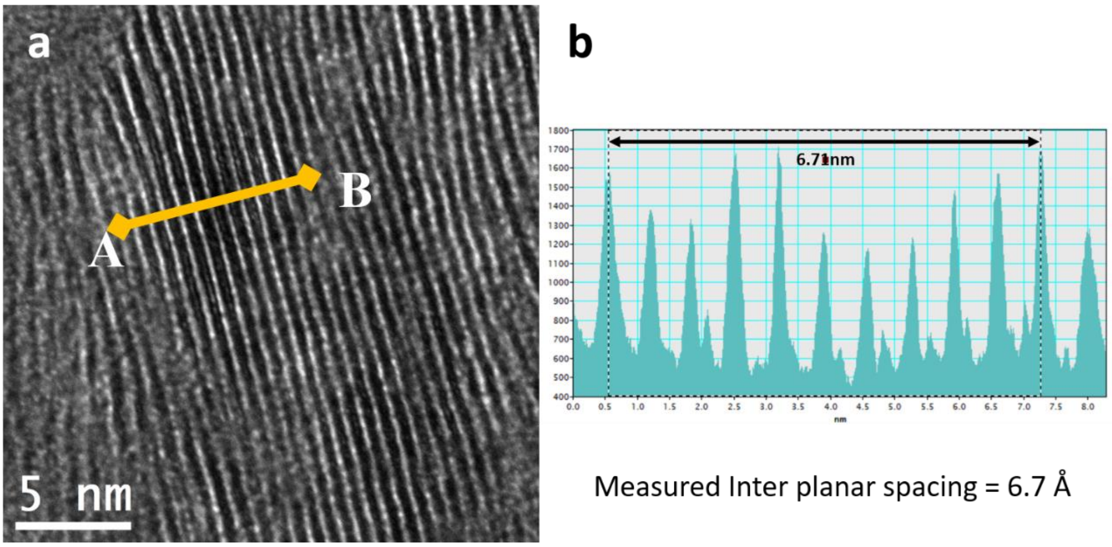

Figure S1. TEM image showing the fringe contrast coming from parallel sets of MoS_2 planes (a), and (b) the corresponding intensity profile across the line marked AB.

EDX Characterization:

Cu presence in the EDX spectrum can be observe (Cu peak close to 8 keV is clearly visible), as there is no other source of Cu in the system.

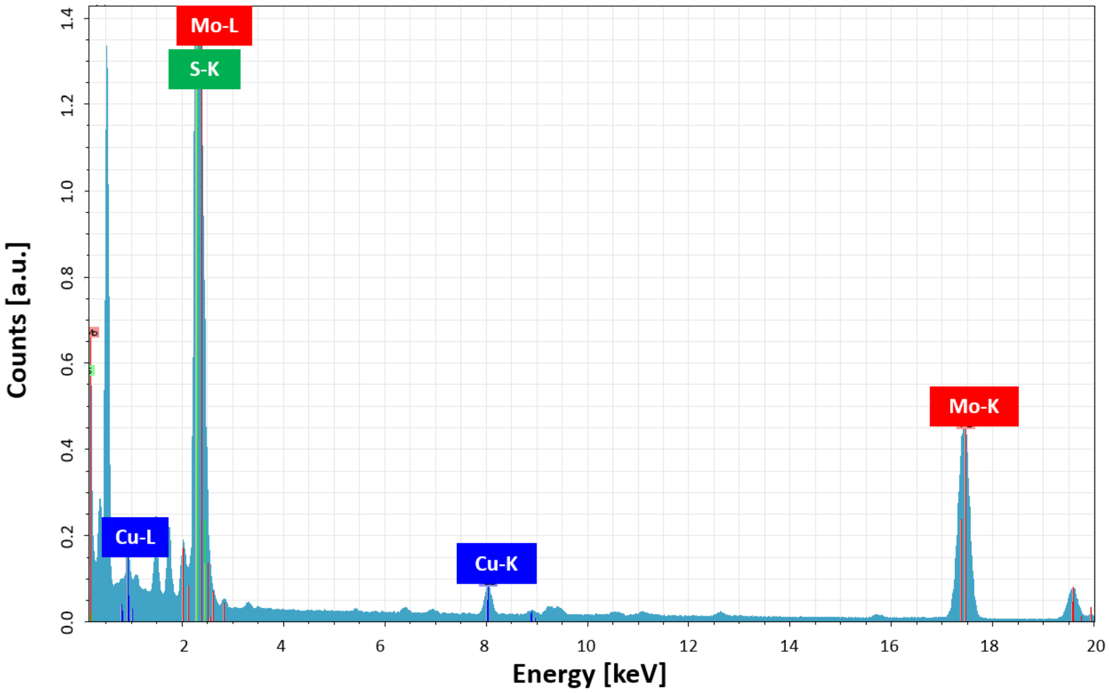

Figure S2. EDS spectrum from energy dispersive spectra mapping of sulfur, molybdenum and copper. The sample was mounted on a Mo grid.

Thermal conductivity measurements

The thermal conductivity of MoS_2 before and after Cu intercalation was calculated with **Eq. S1** - based on the measured thermal and power coefficients of the Raman scattering as recorded on the A_{1g} mode. [1-6]

Eq. S1 $$k = \frac{\chi^T_{A_{1g}}}{2\pi h \chi^P_{A_{1g}}}$$

Where h is the sample thickness, $\chi^P_{A_{1g}} = \frac{\delta\omega_{A_{1g}}}{\delta P}$ is the first order (linear) shift in the A_{1g} mode frequency with respect to the illumination power (**figure S3**) and $\chi^T_{A_{1g}} = \frac{\delta\omega_{A_{1g}}}{\delta T}$ is the first order shift in A_{1g} mode frequency with respect to the temperature (**Figure 1f**). Here the sample thickness is 200 nm, the first order thermal coefficient value is 0.0127 and 0.0091 [cm K]$^{-1}$ (for the bare-Mos_2 and Cu-Mos_2 respectively) and the power coefficient for type of

samples (after averaging measurements from several samples) is ~ 1.4 [cm mW]$^{-1}$. The thermal conductivity measured for MoS_2 is 6.8 for bare VA-MoS_2 and 5.1 W/mK after intercalation of copper.

Calibration of photocurrent:

In order to achieve uniform illumination, the incident light source was kept at beam diameter of ~1cm, where the device diameter is 40 µm. More accurate calibration of the photocurrent as recorded with monochromatic light (**Figure 4d**) was performed by measuring the total power of the light source together with the beam shape with a Spiricon SP620 laser beam profiler. The gaussian power distribution of the beam is accounted for by normalizing actual power as the integrated power density over the device area at the center of the beam.

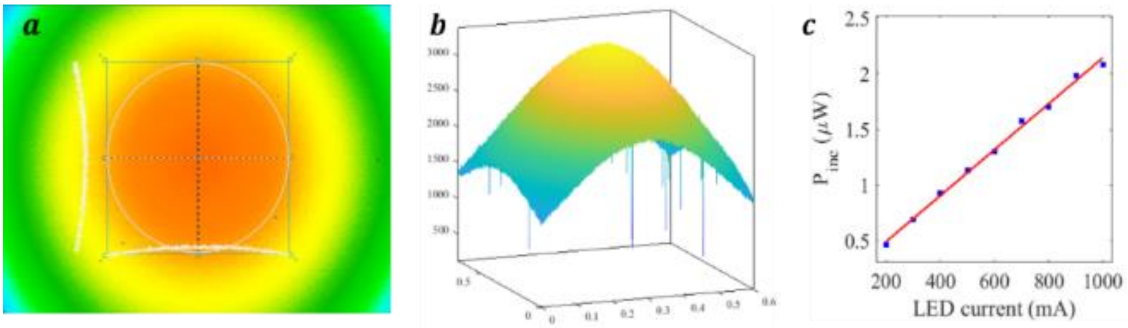

Figure S3. Beam profile (a) 2D and 3D (b) image of the power distribution of a 850 nm LED. (c) The effective incident power on the device under test, as function of driving current (blue dots).

Spectral measurements and calibration:

Reflection measurements:

Reflection spectra were obtained using a Thermo Fisher Scientific Nicolet iS50R FT-IR and a Nicolet ContinuµM FT-IR microscope. Spectral range was set to 9,000-27,000[cm^{-1}] using a Quartz-Halogen (27000-2000 cm^{-1}) source, a Quartz beam splitter and a Silicon detector. Aperture size was set to fit the devices aperture. A 15× objective lens was used to focus light and collect spectra from both a silver mirror as the background and the devices as the sample.

Responsivity measurements:

The photocurrent spectra were obtained by electrically connecting the measured devices to the external detector socket of the instrument, replacing the instrument’s detector. Spectral range was set to 9,000-27,000[cm^{-1}] using a Quartz-Halogen (27000-2000 cm^{-1}) source and a

Quartz beam splitter was used. To obtain the spectral irradiance at the measured device, the single-beam spectrum of the Quartz-Halogen lamp was measured independently using the FTIR DTGS detector, which has a fairly flat responsivity. Due to lack of the specific responsivity spectrum of the used DTGS detector imbedded in the FT-IR instrument, only the qualitative shape of the Quartz-Halogen source was extracted by fitting the single-beam result to Planck's black-body law with a temperature of 1800 K. After normalizing the irradiance obtained from the Planck model by its peak, the ratios of the power emitted at each wavelength remain constant. Next, the measured photocurrent spectrum was normalized to the source's black body curve, achieving the qualitative shape of the spectral responsivity curve. Finally, the qualitative spectral responsivity curve was normalized to the value found at 850 nm and then matched to the responsivity value obtained with a 850 nm calibrated LED, independently measured as described in the photocurrent measurements in the article supplementary.

Time response

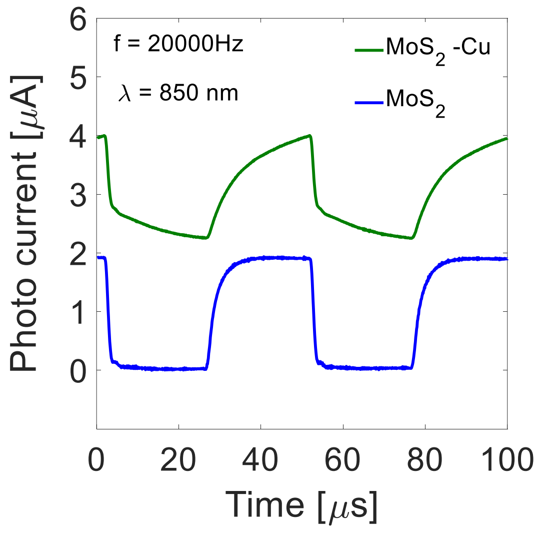

Figure S4. Time dependent photoresponse of VA-MoS_2 (blue), and Cu intercalated MoS_2 devices (green) illuminated at 850nm, P_{in} =1.5μW, under reverse bias of -2V. Photocurrent as a function of time. One response cycle at 20 kHz.

Electrical transport properties

I-V curves of the devices with and without Cu intercalation:

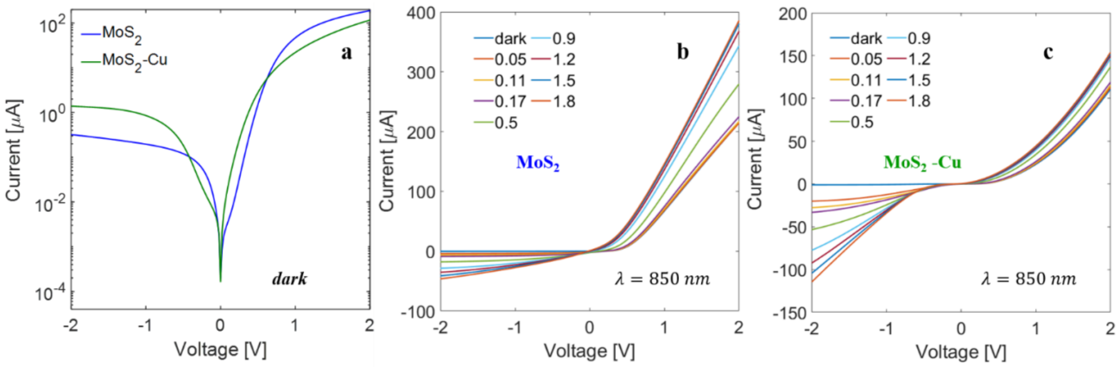

Figure S5. Comparison I-V of the two types of devices with and without Cu intercalation. (a) I-V curve in log scale of the bare-MoS2 (blue) and the Cu-MoS2 (green) at dark. (b) I-V photoresponse curve in linear scale of the bare-MoS2 at different power illuminations (µW). in 850 nm (c) I-V photoresponse curve in linear scale of the Cu-MoS2 at different power illuminations (µW) in 850 nm.

References

1. Yan, R., et al., *Thermal conductivity of monolayer molybdenum disulfide obtained from temperature-dependent Raman spectroscopy.* ACS nano, 2014. **8**(1): p. 986-993.
2. Sahoo, S., et al., *Temperature-dependent Raman studies and thermal conductivity of few-layer MoS2.* The Journal of Physical Chemistry C, 2013. **117**(17): p. 9042-9047.
3. Bae, J.J., et al., *Thickness-dependent in-plane thermal conductivity of suspended MoS2 grown by chemical vapor deposition.* Nanoscale, 2017. **9**(7): p. 2541-2547.
4. Hu, Z., et al., *Temperature dependent Raman and photoluminescence of vertical WS2/MoS2 monolayer heterostructures.* Science Bulletin, 2017. **62**(1): p. 16-21.
5. Lanzillo, N.A., et al., *Temperature-dependent phonon shifts in monolayer MoS2.* Applied Physics Letters, 2013. **103**(9): p. 093102.
6. Wang, R., et al., *Measurement of the thermal conductivities of suspended MoS 2 and MoSe 2 by nanosecond ET-Raman without temperature calibration and laser absorption evaluation.* Nanoscale, 2018. **10**(48): p. 23087-23102.
